# The lesion site of organophosphorus-induced central apnea and the effects of antidotes

**DOI:** 10.1038/s41598-023-47745-x

**Published:** 2023-11-21

**Authors:** Kazuhito Nomura, Eichi Narimatsu, Yoshihiko Oke, Yoshitaka Oku

**Affiliations:** 1 Department of Physiology, Hyogo Medical University, 1-1, Mukogawa-Cho, Nishinomiya-Shi, Hyogo-Ken, 663-8501 Japan; 2 Department of Emergency Medicine, Sapporo Medical University, Sapporo-Shi, 064-8543 Japan

**Keywords:** Inhibition-excitation balance, Excitability, Neurological disorders, Neurotoxicity syndromes

## Abstract

Organophosphorus poisoning kills individuals by causing central apnea; however, the underlying cause of death remains unclear. Following findings that the pre-Bötzinger complex impairment alone does not account for central apnea, we analyzed the effect of paraoxon on the brainstem-spinal cord preparation, spanning the lower medulla oblongata to phrenic nucleus. Respiratory bursts were recorded by connecting electrodes to the ventral 4th cervical nerve root of excised brainstem-spinal cord preparations obtained from 6-day-old Sprague–Dawley rats. We observed changes in respiratory bursts when paraoxon, neostigmine, atropine, and 2-pyridine aldoxime methiodide were administered via bath application. The percentage of burst extinction in the paraoxon-poisoning group was 50% compared with 0% and 18.2% in the atropine and 2-pyridine aldoxime methiodide treatment groups, respectively. Both treatments notably mitigated the paraoxon-induced reduction in respiratory bursts. In the neostigmine group, similar to paraoxon, bursts stopped in 66.7% of cases but were fully reversed by atropine. This indicates that the primary cause of central apnea is muscarinic receptor-mediated in response to acetylcholine excess. Paraoxon-induced central apnea is hypothesized to result from neural abnormalities within the inferior medulla oblongata to the phrenic nucleus, excluding pre-Bötzinger complex. These antidotes antagonize central apnea, suggesting that they may be beneficial therapeutic agents.

## Introduction

Organophosphates (OP) are chemically synthesized poisons, except for those synthesized by certain cyanobacteria (e.g., Guanitoxin), and are used in insecticides and chemical weapons^1,2^. Nearly 200,000 cases of organophosphorus poisoning are reported annually worldwide, with a mortality rate of about 15%^3–6^. OP binds to the serine hydroxyl group in the enzymatically active site of acetylcholinesterase (AChE), leading to enzyme inhibition. This inhibition results in various abnormalities in both humans and animals due to an excess of acetylcholine (ACh)^1,4,7,8^. OP binds to AChE via phosphorylation, creating an irreversible inhibitory effect. Oxime agents can break this OP-AChE bond, restoring AChE activity and reducing excess acetylcholine. However, the OP-Ach bond undergoes ionization through a time-dependent dealkylation reaction. This leads to an ”aging” phenomenon, after which it no longer reacts with oxime^7^. Excessive ACh, being a crucial neurotransmitter in the brain, can lead to neuronal hyperactivity, resulting in delirium, seizures, and respiratory arrest^1,4,7,8^. Respiratory arrest immediately after OP exposure can be peripheral, in which the neuromuscular junction is affected, or central, in which the respiratory center of the brain is affected^1,4,7–10^. Sontakke et al. and Sungur et al. found that respiratory failure was the most important cause of OP poisoning deaths^8,11^. Jayawardane et al. reported that patients exposed to OP had central apnea^12^. Giyanwani et al. reviewed publications on OP-induced respiratory failure publications from 2001 to 2016, revealing that approximately 25% of patients needed ventilation. They concluded that OP-induced respiratory failure is mainly a central mechanism and a significant contributor to acute mortality^13^. Central apnea is a fatal condition, necessitating a more scientific and efficacious clinical approach. Despite numerous studies on OP-induced central apnea, its underlying mechanisms remain elusive. Consequently, treatment and prevention methods differ greatly among experts, leading to ongoing confusion in this field^1,4,7,8,13–17^.

The respiratory rhythm in unconscious mammals originates from the pre-Bötzinger complex (PBC), located in the ventral lateral region of the lower medulla oblongata^18^. The rhythmic electrical excitation events in the PBC are termed "respiratory bursts". These bursts are responsible for transmitting signals to the diaphragm and upper airway muscles, generating inspiratory movements, and maintaining upper airway patency. The characteristics of these respiratory bursts, including amplitude, duration, and frequency, correlate with actual respiratory movements, classifying them as fictive breathing^18–22^. The PBC serves as the core of respiratory control, and its impairment leads to central apnea. Various diseases manifesting abnormal respiratory rhythms suggest dysfunction or pathogenic abnormalities within the PBC^23–25^. A significant portion of PBC activity is modulated by glutamate. However, the presence of ACh receptors in the PBC also plays a regulatory role in shaping the respiratory rhythm^26–29^. Consequently, excess ACh may lead to aberrant PBC activity. Therefore, the OP-induced central apnea was hypothesized to be caused by the cessation of respiratory burst excitation of the PBC.

Prior studies have demonstrated that direct OP injection into the animal brainstem or retrograde OP perfusion from the aorta into the central nervous system (CNS) suppresses respiratory movements and phrenic nerve firing^10,30–33^. In these studies, both in vivo models involving whole animal bodies and in situ models, such as working heart brainstem preparation (WHBP), were employed. These experimental approaches offer the advantage of observing the reactions under physiologically relevant conditions. Nevertheless, the functions and mechanisms of the respiratory center, including PBC, remain elusive. Moreover, PBC synchronizes its functions through signal exchange with peripheral organs as well as with other CNS regions, including the pons, midbrain, cerebellum, and cerebrum. Hence, the analysis of the outcomes from these experimental approaches is intricate and challenging. For instance, ACh binds to the M2 muscarinic receptor in the heart, leading to a decrease in pulse rate and binding to the M1 and M3 muscarinic receptors in vascular smooth muscle, resulting in reduced peripheral vascular resistance. This sequence of events can lead to diminished blood flow in the brainstem and subsequent reduction in the functionality of the respiratory center. In experiments involving the WHBP, cerebral circulation was upheld through extracorporeal circulation, yet these preparations included the pons^10,31–33^. The pons houses pneumotaxic and apneustic centers, influencing PBC and phrenic nucleus activity, but their regulatory mechanisms and functions remain complex and unclear^34–36^. Furthermore, when investigating the impact of OP administration on specific localized brain areas, OP must be introduced via a slender capillary inserted into the brain, which inevitably results in physical brain damage.

Moreover, studies on OP and central apnea have varied widely in terms of animal type, drug type, drug dosage, and experimental methods, and none have directly examined the electrical activity of PBC. To remove these obstacles, it is necessary to choose a "small" experimental model consisting of fewer organs and tissues. The "smallest" experimental model that allows electrophysiological and pharmacological studies of respiratory bursts spontaneously generated from PBC is the "respiratory slice" of a thin transverse section preparation (thickness of approximately 500 μm) containing PBC. We have used it in the past to test the widely held hypothesis that OP directly stops the electrical activity of PBC^37^. The study revealed that paraoxon (Pox; O, O-diethyl O-(4-nitrophenyl) phosphate) reduced the burst amplitude of both the PBC and hypoglossal nucleus but did not stop the bursts. This was inconsistent with the previous hypothesis that patients with OP poisoning die from central apnea due to PBC failure.

These findings suggest that OP-induced central apnea cannot be solely attributed to abnormalities in PBC activity. In fact, the study showed a reduction in PBC amplitude of approximately 20%. While this reduction may contribute to central apnea, it is thought to be caused by abnormalities in other neural regions, as central apnea refers to a complete cessation of respiratory bursts. It is possible that blockage occurs in the pathway for respiratory bursts generated in the PBC to reach the C4 nerve. Based on these observations, we propose a new hypothesis that OP-induced central apnea is caused by abnormalities in the neural region from the lower medulla oblongata to the phrenic nucleus, excluding the PBC. To test this hypothesis, we used a brainstem-spinal cord preparation, also known as an "en bloc" preparation, which was developed by Suzue in 1984^19^. This preparation allows for the extraction of the medulla oblongata to the upper cervical cord in an intact state, and respiratory bursts are stable for long periods of time and can be observed when the ventral C4 nerve is held in suction with a glass capillary electrode^19–22^.

Parathion is an extremely toxic insecticide that has been responsible for numerous fatalities in the past. This compound undergoes an oxidative desulfurization reaction in animals, resulting in the formation of Pox, which is even more toxic than parathion due to its potent inhibition of the AChE^38^. It has been noted that in recent years, the use of parathion and Pox as pesticides has decreased due to the recognition of their harmful effects. Conversely, Pox has been utilized in research as a substitute for organophosphorus chemical agents, which are challenging to acquire and manage^37,39–42^. Therefore, Pox was used in this study as a representative drug for OP.

Furthermore, although two antidotes for OP, atropine and 2-pyridine aldoxime methiodide (2-PAM), are used in clinical practice, there is insufficient evidence to show that they are effective in OP-induced central apnea. Atropine, a muscarinic receptor selective antagonist, does not act on nicotinic receptors and may be less effective. Atropine can induce tachycardia by inhibiting muscarinic receptors in the heart. However, it can also cause bradycardia by obstructing M1 muscarinic receptors in the sympathetic ganglia^43,44^. One study also reported that atropine induced lethal arrhythmias in an animal model of OP poisoning^45^. Those circulatory instabilities can lead to decreased cerebral blood flow and brainstem dysfunction. The large number of variable physiological factors and the complexity of their interactions make the therapeutic effect of atropine on OP-induced central apnea challenging to understand. 2-PAM is a clinically available oxime agent that possesses the capability to break the OP-AChE bond, but it is plagued by the limitation that it rarely penetrates the blood–brain barrier (BBB)^46^. It remains unclear if 2-PAM is intrinsically ineffective for OP-induced central apnea or if its limited penetration through the BBB compromises its efficacy. The brainstem-spinal cord preparation lacks a BBB, heart, and signaling to peripheral organs, making it ideal for studying the fundamental effects of these antidotes. Consequently, we sought to identify primary lesions from OP-induced central apnea and evaluate antidote efficacy.

## Results

In a single experiment per preparation, a total of 50 rats were used. As outlined in the protocol section, drugs were delivered through bath application. The artificial cerebrospinal fluid (ACSF) perfused into the chamber was discarded without reuse. As mentioned in the recording and analysis section, respiratory burst parameters, including amplitude, duration, and frequency, were averaged every minute. The 20th-minute average was deemed the "negative control value." Subsequent averages were expressed as percentages relative to these control values. The 60th-minute ratios were considered the "resultant values," and statistical comparisons were made across the groups.

### Naïve group (n = 8, no drug application)

Normal ACSF (drug-free ACSF, see Preparation section) was continuously perfused for 60 min (Table 1). The resultant values of the amplitude, duration, and frequency in the naïve group (n = 8) were 50.3% ± 17.6, 98.1% ± 15.3, and 109.0% ± 19.9, respectively. Burst elimination was not observed in any rat in this group.

**Table 1:** Experimental design and drugs administered.

Group	Control (20 min)	Drug application (20 min)	Washout (20 min)
Naïve	Normal ACSF	Normal ACSF	Normal ACSF
Pox		Paraoxon 10 μM	
Pox + atr		Paraoxon 10 μM + atropine 1 μM	
Pox + pam		Paraoxon 10 μM + 2-PAM 100 μM	
Neo		Neostigmine 100 μM	
Neo + atr		Neostigmine 100 μM + atropine 1 μM	

*ACSF* artificial cerebrospinal fluid, *Pox* paraoxon, *atr* atropine sulfate, *Neo* neostigmine bromide, *pam* 2-PAM.

### Pox-poisoning group (n = 10, Pox 10 μM)

**Figure 1:**
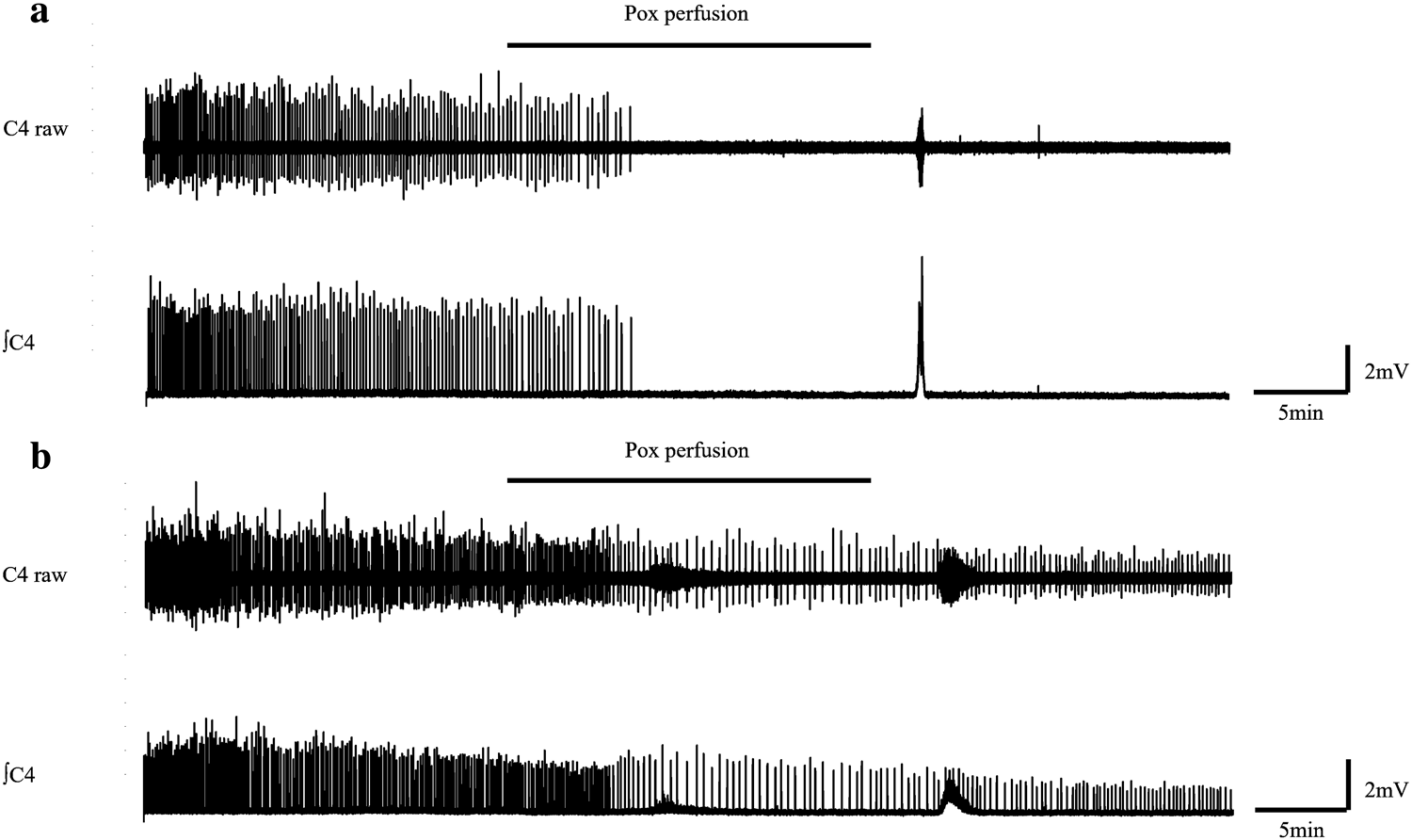
(**a**) Illustration of respiratory burst arrest induced by Pox. (**b**) Illustration of decreased respiratory burst frequency due to Pox. *Pox* Paraoxon, *C4* Respiratory burst waveform originating from the 4th cervical spinal nerve roots, *∫C4* Integral waveform of C4, ━ Pox perfusion.

**Figure 2:**
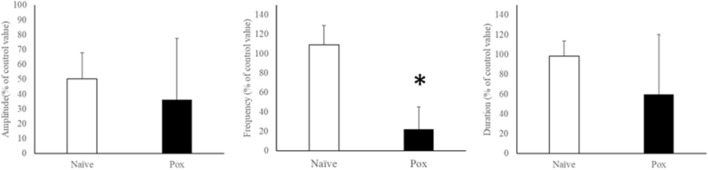
Effects of paraoxon administration. Comparison of the amplitude, frequency, and duration values in the naïve and Pox groups. *Statistical significance was determined using Welch's t-test (p < 0.05). *Pox* paraoxon.

Normal ACSF was changed to Pox-containing ACSF at the 20th minute and perfused back to normal ACSF at the 40th minute (Table 1). The resultant values of the amplitude, duration, and frequency in the Pox-poisoning group were 27.5% ± 28.5, 59.7% ± 60.4, and 22.1% ± 22.9, respectively, with complete disappearance of bursts in five preparations (50.0%) (Fig. 1a). Even in preparations in which bursts did not disappear, the frequency of respiratory bursts tended to decrease (Fig. 1b). Using Welch's t-test, no difference was observed in the amplitude and duration between the naïve and Pox-poisoning groups; however, there was a significant difference in the frequency (naïve vs. poisoning group, 109.0% ± 19.9 vs. 22.1% ± 22.9; p < 0.05) (Fig. 2).

### Pox-treatment group with atropine (n = 6, Pox 10 μM, atropine 1 μM)

**Figure 3:**
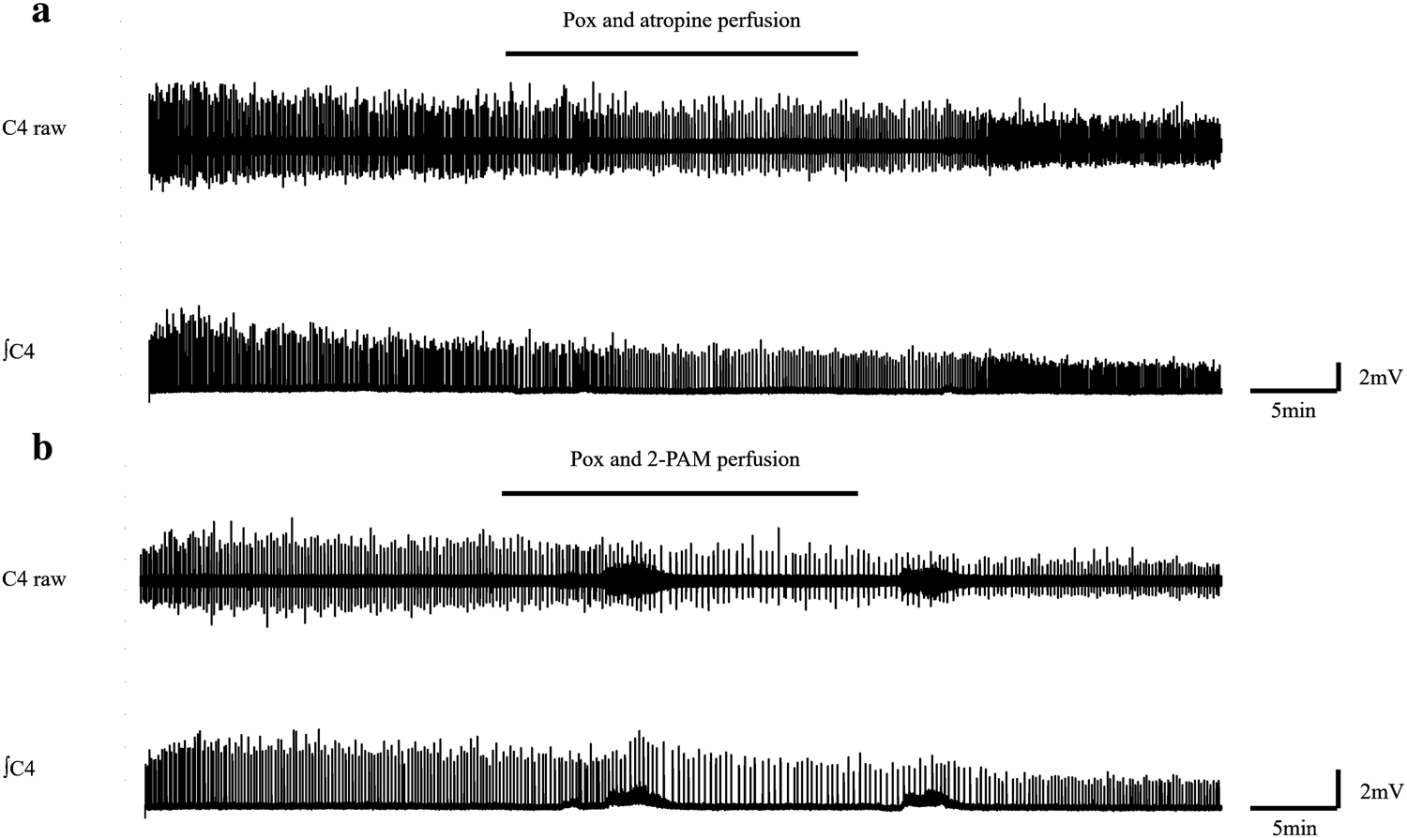
(**a**)Depiction of the waveform during concurrent administration of Pox and atropine. (**b**) Another representation of the waveform under simultaneous Pox and 2-PAM administration. Both antidotes, atropine and 2-PAM, counteracted the central apnea triggered by Pox. *Pox* Paraoxon, *C4* Respiratory burst waveform originating from the 4th cervical spinal nerve roots, *∫C4* Integral waveform of C4, ━ Pox and antidotes (atropine or 2-PAM) perfusion.

**Figure 4:**
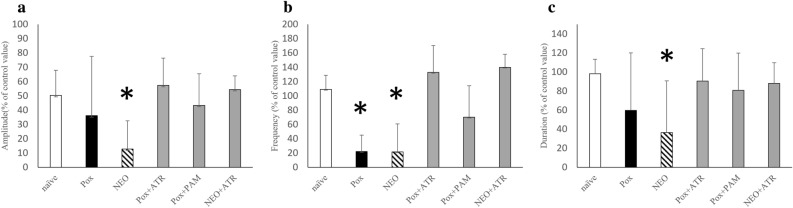
Effects of Pox and Neo administration and pam and atr administration. The Naïve, Pox, Neo, Pox + atr, Pox + pam, and Neo + atr groups were compared in terms of (**a**) amplitude, (**b**) frequency, and (**c**) duration. *Statistical significance was determined using a Bonferroni correction (p < 0.05). *Pox* paraoxon, *Neo* neostigmine bromide, *atr* atropine sulfate, *pam* 2-PAM.

Normal ACSF was changed to Pox and atropine containing ACSF at the 20th minute and perfused back to normal ACSF at the 40th minute (Table 1). The resultant values of the amplitude, duration, and frequency in the Pox-treatment group with atropine (n = 6) were 57.3% ± 19.1, 90.5 ± 34.2, and 132.8% ± 37.6, respectively. No preparations exhibited complete burst disappearance (0%) (Fig. 3a). Using Bonferroni correction for multiple comparisons among the naïve, Pox-poisoning, and Pox-treatment groups, there were no significant differences in burst amplitude and duration across all groups. The frequency of bursts between the naïve and Pox-treatment groups also did not differ significantly (109.0% ± 19.9 vs. 132.8% ± 37.6) (Fig. 4).

### Pox-treatment group with 2-PAM (n = 11, Pox 10 μM, 2-PAM 100 μM)

Normal ACSF was changed to Pox and 2-PAM containing ACSF at the 20th minute and perfused back to normal ACSF at the 40th minute (Table 1). The resultant values of the amplitude, duration, and frequency in the Pox-treatment group with 2-PAM (n = 11) were 38.9% ± 20.1, 77.8% ± 40.5, and 70.2% ± 44.0, respectively. The bursts completely disappeared in two preparations (18.2%) (Fig. 3b). Using Bonferroni correction, comparisons among the naïve, Pox-poisoning, and Pox-treatment groups revealed no significant differences in burst amplitude and duration across all combinations. The burst frequency between the naïve and Pox-treatment groups also showed no significant difference (109.0% ± 19.9 vs. 70.2% ± 44.0) (Fig. 4).

### Neostigmine-poisoning group (n = 9, neostigmine 100 μM)

**Figure 5:**
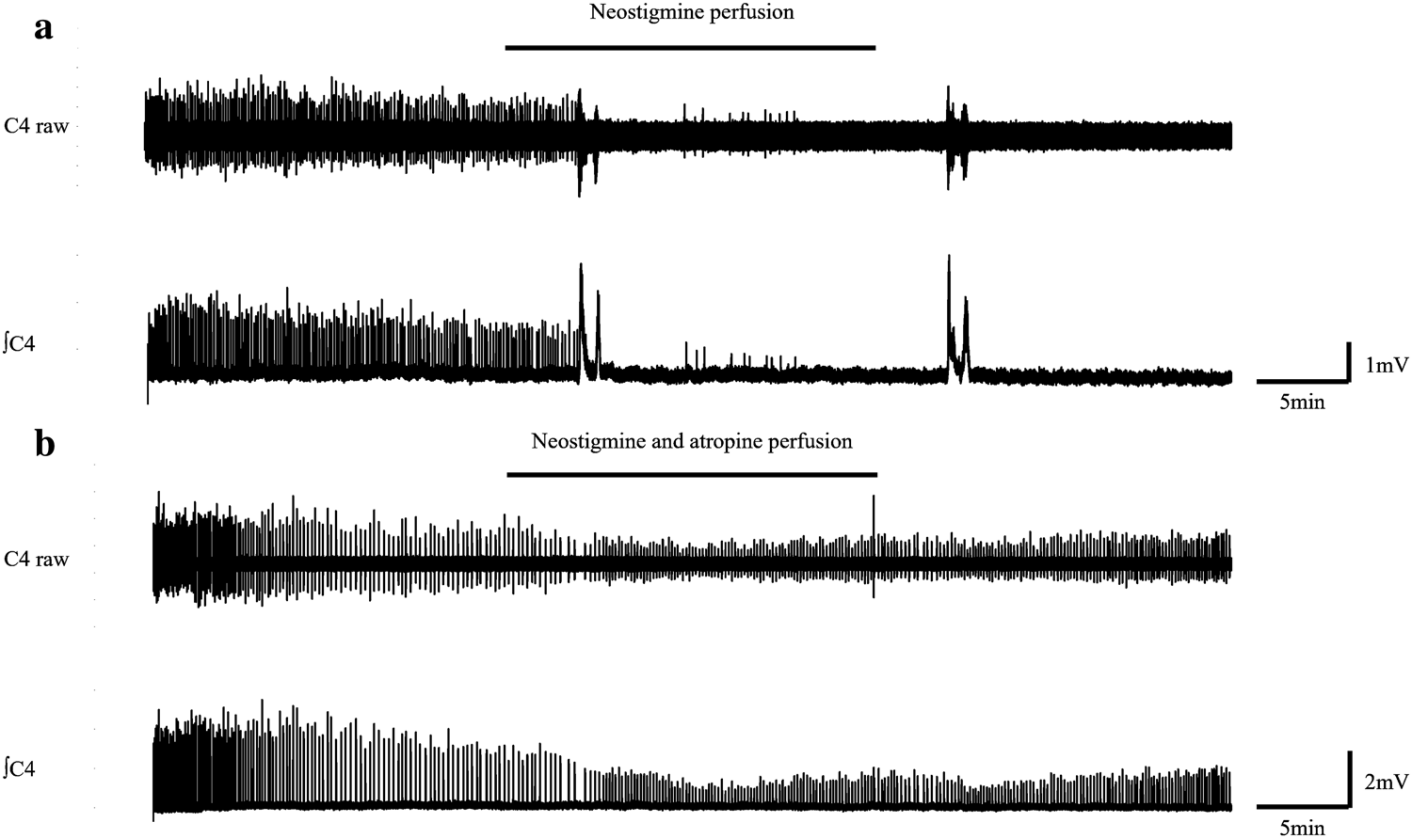
(**a**) Illustration of respiratory burst arrest induced by neostigmine. (**b**) Depiction of the waveform during concurrent administration of neostigmine and atropine. *C4* Respiratory burst waveform originating from the 4th cervical spinal nerve roots, *∫C4* Integral waveform of C4, ━ Neostigmine (and atropine) perfusion.

Normal ACSF was changed to neostigmine containing ACSF at the 20th minute and perfused back to normal ACSF at the 40th minute (Table 1). The resultant values of the amplitude, duration, and frequency in the neostigmine-poisoning group were 12.9% ± 19.7, 36.5% ± 54.3, and 21.5% ± 39.3, respectively. In this group, the bursts completely disappeared in six preparations (66.7%) (Fig. 5a). Using Welch's t-test, the amplitude, duration, and frequency were significantly different (naïve vs. neostigmine poisoning: 50.3% ± 17.6 vs. 12.9% ± 19.7, 98.1% ± 15.3 vs. 36.5% ± 54.3, and 109.0% ± 19.9 vs. 21.5% ± 39.3, respectively; p < 0.05) (Fig. 4).

### Neostigmine-treatment group (n = 6, neostigmine 100 μM, atropine 1 μM)

Normal ACSF was changed to neostigmine and atropine containing ACSF at the 20th minute and perfused back to normal ACSF at the 40th minute (Table 1). The resultant values of the amplitude, duration, and frequency in the neostigmine-treatment group with atropine (n = 6) were 54.4% ± 9.6, 88.1% ± 21.7, and 140.0 ± 18.3, respectively. There were no preparations for which the bursts disappeared completely (0%) (Fig. 5b).

Using the Bonferroni correction, comparisons among the naïve, neostigmine poisoning, and neostigmine treatment groups revealed no significant differences between the naïve and neostigmine treatment groups;amplitude (50.3% ± 17.6 vs. 54.4% ± 9.6), duration (98.1% ± 15.3 vs. 88.1% ± 21.7), and frequency (109.0% ± 19.9 vs. 140.0 ± 18.3)(Fig. 4).

## Discussion

In this study, respiratory bursts ceased in half of the subjects in the Pox-poisoning group, and there was a significant reduction in burst frequency. This effect was countered by the co-administration of antidotes, either atropine or 2-PAM. The study also demonstrated that neostigmine, a carbamate AChE inhibitor with a mechanism distinct from organophosphorus, elicited a response akin to that of Pox. Notably, atropine fully counteracted the toxic effects of both Pox and neostigmine. These findings suggest that Pox triggers central apnea. The affected sites are within the scope of our "en bloc" preparation, with the underlying cause being excessive ACh.

OP is a highly lethal poison and has been implicated in central apnea as a cause of death^1,4,7,8,10–13^. However, the lack of research on OP-induced central apnea makes it difficult to formulate evidence for its treatment and prevention. OP has caused numerous casualties and illnesses in the past, including the 1995 sarin gas attack on a subway in Japan^47^ and the 2013 Syrian civil war^48^. Similar incidents may occur in various parts of the world in the future, owing to conflicts and terrorism. Research on the mechanism of central apnea, such as that in this study, is of great medical significance because numerous casualties may occur unless scientifically valid treatment measures are established.

As mentioned in the Introduction, the rejection of the "PBC-alone failure hypothesis" provided us with a new hypothesis. The brainstem-spinal cord preparation used was an intact extraction of the neural circuit from the inferior medulla oblongata (at a height that includes the PBC) to the C4 nerve. During normal ACSF perfusion, while the C4 was being held in suction with a glass electrode, the respiratory burst excitation generated from the en bloc preparations was recorded over time. In this setup, if a drug whose effects are to be verified is mixed with normal ACSF, the drug effects can be observed electrophysiologically. In our setup, the pons and peripheral organ signals are disconnected, and with the absence of cerebral blood flow, hemodynamic effects are irrelevant. This preparation consistently produces respiratory bursts over extended periods. It serves as an intermediate experimental model, is "larger" than the "respiratory slice," but "smaller" than in situ WHBP or in vivo whole-body models. This makes it ideal for hypothesis testing by minimizing potential causes of respiratory rhythm disturbances. The brainstem-spinal cord preparations we employ can encompass various organs based on the experimental objective (e.g., pons, cerebellum, entire spinal cord, diaphragm, peripheral nerves of extremities). Therefore, it offers flexibility to integrate additional organs, paving the way for studies with "larger" experimental models^21^.

The mammalian physiological respiratory rhythm is triphasic, comprising inspiration, post-inspiration, and late expiration, with the pons playing a role in its regulation. When the pons of WHBP is severed, it is observed that respiratory bursts become unstable and can easily vanish^49^. Conversely, in brainstem-spinal cord preparations, respiratory bursts consistently persist for over an hour, even post-pons removal^50^. Moreover, the pons transmits inhibitory signals to the PBC. Thus, brainstem-spinal cord preparations with an intact pons exhibit a reduced frequency of respiratory bursts. This frequency escalates once the pons is removed^51^. This study aimed to ascertain if OP curtails respiratory bursts. If the bursts are inherently infrequent, distinguishing whether a decrease is attributed to OP exposure or mere chance becomes challenging. Hence, it was necessary to sever the pons.

The findings from our previous study^37^, when combined with those of the current study, suggest that the primary lesion responsible for OP-induced central apnea spans from the lower medulla oblongata to the phrenic nucleus, excluding PBC. However, as this research solely examined changes in respiratory bursts from the C4 nerve, it does not pinpoint specific abnormalities within that region. Pox is a representative OP that exerts potent inhibitory effects on AChE and produces excess ACh. The following three mechanisms may be responsible for the excess ACh causing respiratory burst arrest: (1) High concentrations of ACh derivatives in the phrenic nucleus reportedly reduce the phrenic burst amplitude^52^. Furthermore, attenuation of the respiratory bursts of PBC are reported following Pox administration^37^. This combination of PBC suppression and amplitude attenuation in the phrenic nucleus may have led to the burst disappearance. (2) At the C1–C2 level of the spinal cord, there is a neural region called the "high cervical respiratory neuron group" (HCRG) that innervates and projects to the PBC^53^. HCRG can modify the electrical activity of PBC; thus, HCRG destruction can cause the disappearance of PBC's respiratory burst^54^. Abnormal seizure-like excitation in the spinal cord is transmitted to the medulla oblongata, which attenuates the normal respiratory bursts^55^. Thus, the respiratory bursts of the PBC may have stopped secondary to the Pox-induced dysfunction of the upper cervical neuron group. (3) The pathway from the PBC to the phrenic nucleus is not a simple transmission by monosynaptic connections but a complex one mediated by a group of interneurons^56^. A part of this group of interneurons may have been disrupted by Pox, resulting in the disruption of neurotransmission and loss of respiratory burst excitation from C4.

Furthermore, we evaluated the possibility that Pox causes central apnea by exerting effects other than AChE inhibition. A group was established to receive neostigmine, a carbamate AChE inhibitor, with a mechanism different from that of OP. It was found that neostigmine abolished the respiratory burst, which was antagonized by atropine. These results indicate that Pox-induced respiratory burst loss is caused by an excessive ACh state. Another potential explanation is that elevated ACh concentrations could antagonize nicotinic receptors, thereby suppressing the nicotinic receptor-mediated excitation of PBC neurons and causing respiratory bursts to vanish^58^. However, atropine, a specific antagonist of muscarinic receptors, fully counteracts the toxic effects of both OP and neostigmine. This implies that while nicotinic receptor-mediated responses may play a role in central apnea, their contribution is likely minimal.

The antidotes have a history of being approved based solely on their pharmacological mechanisms of action, without clinical trials being conducted with them as therapeutic agents. In particular, the clinical efficacy of 2-PAM is controversial^1,4,7,8,14–17^. However, the present study clearly showed that 2-PAM antagonizes OP-induced central apnea. This was a significant achievement because it demonstrated the benefits of 2-PAM. Nevertheless, 2-PAM has limited penetration through the BBB^15–17^.

In this study, 2-PAM was chosen primarily because it is the most recognized oxime with anti-OP properties, and the absence of BBB in brainstem-spinal cord preparations made its penetrance a non-issue. For oxime agents to be effective in genuine central apnea, they must effectively penetrate the BBB. Fortunately, several promising oxime candidates have been developed thus far^15^.

No preparations in the atropine group and 18.2% of preparations in the 2-PAM group had respiratory burst arrest. Therefore, 2-PAM is inferior to atropine in terms of effectiveness. This could be due to the following reasons: (1) atropine and 2-PAM exhibit different mechanisms of action, and (2) atropine has no inhibitory effect on nicotinic receptors. Atropine is a nonspecific competitive inhibitor of muscarinic receptors that can exert an immediate antagonistic effect against increasing ACh. In contrast, 2-PAM restores the enzymatic activity of AChE by cleaving the linkage between AChE and OP^15–17^. Because it accelerates the breakdown of ACh, causing lower ACh concentrations in the tissues, the onset of its effect is slower than that of atropine. We estimated that approximately 20% of the preparations remained toxic to paraoxon.

Muscarinic receptor-mediated cell excitation leads to cellular apoptosis via elevation of intracellular calcium due to calcium release from the endoplasmic reticulum. However, nicotinic receptor-mediated stimulation inhibits neuronal apoptosis^57^, and more neurons may have survived because of the remaining nicotinic receptor-stimulating effect. At a minimum, atropine administration fully counteracts the toxicity of Pox and neostigmine; therefore, central apnea is predominantly a muscarinic receptor-mediated response. This is the first study to have experimentally reproduced and electrophysiologically analyzed OP-induced central apnea using brainstem-spinal cord preparations.

This research is the initial demonstration that the neural region of the lower medulla oblongata, as well as pathways leading to the phrenic nucleus, excluding the PBC, is instrumental in central apnea. Furthermore, it demonstrates that the central apnea phenomenon is antagonized by existing antidotes, indicating that it is an excellent bridging study with implications for therapeutics.

This study has several limitations. First, this study was conducted on rodent en bloc preparations; hence, in the context of humans, a species gap is evident. Second, the respiratory centers of neonatal rodents are immature, suggesting the possibility that they may behave differently from mature animals. Third, as the effects of nicotinic receptor antagonists have not been examined, the intricacies of nicotinic receptor-mediated responses are yet to be elucidated. Fourth, this study primarily pinpointed the general location accountable for respiratory burst arrest, necessitating further research to uncover the underlying detailed mechanisms. To clarify this, we have designed an upcoming study involving a stereotaxic microinjection method to the target sites (such as the HCRG or phrenic nucleus) to determine whether microinjections of Pox alone or Pox along with its antidotes can affect the respiratory burst from the phrenic nerve.

Finally, although rostral regions, such as the pons, are also involved in respiratory rhythm formation, the influence of these regions remains unknown because they were not included in this study.

## Conclusions

Central apnea due to OP is strongly associated with dysfunction in the neural region from the inferior medulla oblongata to the phrenic nucleus, excluding the PBC, which has not received much attention. Two antidotes antagonize central apnea, suggesting that they may be beneficial therapeutic agents.

## Materials and methods

This study was approved by the Animal Experiment Committee of Hyogo Medical University (No: 21-008; 17th June 2021). All the experiments were conducted in compliance with the Hyogo Medical University of Medical Science's Animal Experiment Regulations, relevant laws and regulations in Japan, and prefectural ordinances. This study was conducted in accordance with the ARRIVE guidelines. Fifty 6-day old Sprague–Dawley rats (birth date: day 0) were used in the experiment.

### Preparations

The preparation procedures were based on previous reports^19–22^. The rats were deeply anesthetized with isoflurane at room temperature and no paw reflex was observed in response to pinch stimulation of the hind limbs. Transverse cuts were made at the forehead and the region just above the diaphragm and neural tissues from the cerebral cortex to the thoracic spinal cord were isolated in artificial cerebrospinal fluid (normal ACSF) (NaCl 124 mM, KCl 5.0 mM, CaCl_2_ 2.4 mM, MgCl_2_ 1.3 mM, KH_2_PO_4_ 1.2 mM, NaHCO_3_ 26 mM, and glucose 30 mM, saturated with 95% O_2_ and 5% CO_2_ at 20 °C). Subsequently, the cerebral cortex, cerebellum, and thoracic spinal cord were removed, and the brainstem was cut transversely at the level between the glossopharyngeal and rostral hypoglossal nerves to obtain the brainstem-spinal cord preparation.

### Recording and analysis

The preparations were transferred to the recording chamber immediately after completion.

The chamber was perfused with normal ACSF at 26.5 ± 0.5 °C at a rate of 3–5 ml/min. The fourth cervical nerve root (C4) was identified under a microscope and fixed via suctioning it into a glass capillary electrode. The capillary electrodes were then connected to an amplifier (MEG-6108; Nihon Kohden, Tokyo, Japan) to measure the electrical signals generated during preparation. The obtained signals were bandpass filtered (50–1000 Hz) and input into a computer via an AD converter (PowerLab 4/25 T; ADInstruments, Bella Vista, NSW, Australia). The signals were observed and recorded in real-time using LabChart Pro (version 8.1; ADInstruments, Bella Vista, NSW, Australia). The series of signals were integrated with a time constant of 0.05 s for analysis. The amplitude, duration, and frequency of the bursts obtained were analyzed with reference to a previous study^19–22,37^.

The amplitude was measured as the height from the beginning of the burst to its apex. The duration from the start to the end of the burst was also measured. The amplitudes and durations were averaged per minute, and the frequency was counted directly as the number of bursts per minute.

### Drugs

Paraoxon [Pox; O, O-diethyl O-(4-nitrophenyl) phosphate, an OP and AChE inhibitor], 2-pyridine aldoxime methiodide (2-PAM; an oxime compound), and neostigmine bromide (a carbamate AChE inhibitor) were purchased from Fujifilm Wako Pure Chemical Co., Osaka, Japan. Atropine sulfate (a competitive nonspecific muscarinic receptor antagonist) was purchased from Tokyo Kasei Kogyo Co., Tokyo, Japan. Paraoxon was dissolved in dimethyl sulfoxide (DMSO) to make it water-soluble because it is a liposoluble drug. The DMSO concentration was adjusted to < 0.1% in the ACSF. The rationale for selecting specific drug types and their respective concentrations is outlined below.

*Pox* As highlighted in the Introduction, Pox is frequently employed in OP toxicity studies. Ray et al. conducted a study involving the perfusion of Pox solution into rat brain tissue and demonstrated that AChE inhibition peaked at a Pox concentration of 10 μM^59^. Mirroring their findings, we maintained the same concentration for Pox in this study.

*Neostigmine* Joosen et al., in a study where neostigmine was used as a stand-in for chemical weapons, revealed that perfusing rat brain tissue with 100 μM neostigmine led to a total cessation of AChE activity^60^. Aligning with their research, we set an identical concentration for neostigmine in our study.

*Atropine and 2-PAM* In our prior research^37^, concentrations of 1 μM (for atropine) and 100 μM (for 2-PAM) were shown to exhibit significant antagonism against Pox toxicity. Consequently, these concentrations were retained for this study.

### Protocol

The protocol (Table 1) was determined according to findings reported in previous studies^37,42^. The ACSF was maintained at 26.5 ± 0.5 °C and perfused at a rate of 3 to 5 ml/min using an infusion circuit and a peristaltic pump. The drained ACSF was directly collected in a waste container, ensuring it was not reused or recirculated. Each experiment spanned 60 min and commenced only after verifying the stability and recordability of the respiratory bursts. From start to finish, respiratory bursts were constantly documented. The experimental procedures were segmented into three phases, each lasting 20 min, following the sequence: control, drug application, and washout.

*Control phase* Normal ACSF was perfused for 20 min in all experiments.

*Drug application phase* During this 20-min segment, the regular ACSF was replaced with drug-infused ACSF. The transition was facilitated using a three-way stopcock, and the drug was introduced through a bath application method. In this phase, the group that was administered normal ACSF for the entire 60 min was defined as the naïve group. The group that received 10 μM of Pox was defined as the Pox-poisoning group, and the group that was administered Pox (10 μM) and an antidote drug (1 μM of atropine or 100 μM of 2-PAM) was defined as the Pox-treatment group. Similarly, the group that was administered 100 μM of neostigmine was defined as the neostigmine-poisoning group, and the group that was administered neostigmine (100 μM) and atropine (1 μM) was defined as the neostigmine-treatment group.

*Washout phase* In common with all experiments, normal ACSF was perfused for 20 min.

During the 60-min protocol, the amplitude, duration, and frequency of respiratory bursts were averaged on a per-minute basis. The average burst amplitude, per-minute duration, and burst frequency at the 20th minute were documented as the negative control values. These per-minute mean values for amplitude, duration, and frequency were then transformed into percentages relative to their corresponding negative control values. The percentages of amplitude, duration, and frequency at the concluding point, the 60th minute (20th minute of the washout phase), were designated as the resultant values.

### Statistical analysis

Statistical analyses were conducted, drawing upon methodologies from prior studies^20,22,37,42^. The ratio (%) of the resultant value to the negative control value of each parameter was calculated in each rat. Subsequently, the mean (%) ± standard deviation of the amplitude, duration, and burst frequency ratios of each group was calculated. Statistical comparisons were performed using Welch's t-tests on the results of the naïve and poisoning groups to confirm the effects of Pox and neostigmine. To confirm the effects of the antidotes, statistical comparisons were made between the naïve, poisoning, and treatment groups using multiple comparison tests with the Bonferroni correction. The significance level was set at p < 0.05.

## Data Availability

The datasets generated during and/or analyzed during the current study are available from the corresponding author on reasonable request.
